# Ureteral reconstruction is safe and successful in poorly functioning kidneys

**DOI:** 10.3389/fruro.2025.1593307

**Published:** 2025-09-09

**Authors:** Logan W. Grimaud, Kiran Sury, Matthew Salvino, Austin Livingston, Aaron C. Lentz, Andrew C. Peterson

**Affiliations:** Department of Urology, Duke University School of Medicine, Durham, NC, United States

**Keywords:** ureteral stricture, reconstruction, renal function, dogma, stricture disease

## Abstract

**Objectives:**

Patients with a poorly functioning kidney, defined as less than 20% differential renal function, have historically been considered poor candidates for ipsilateral ureteral reconstruction for stricture. To determine if renal function can be safely preserved in poorly functioning kidneys with ureteral stricture, we evaluated patient outcomes following ureteral reconstruction.

**Methods:**

We conducted a review of 114 adult patients who underwent ureteral reconstructive surgery at our institution between 2013 and 2023. Patients with poorly functioning ipsilateral kidneys were identified by a preoperative renal scan (MAG3 renogram). Variables of interest included patient characteristics, peri/postoperative outcomes, resolution of hydronephrosis, pre/postoperative renal function, and preservation of renal parenchyma.

**Results:**

Of the 8 patients meeting inclusion criteria, 5 underwent bladder elongation psoas hitch (BEPH), 1 ileal ureter, 1 ileal ureter with BEPH, and 1 ureteroureterostomy. Median preoperative differential renal function was 16.0% with a median preoperative serum creatinine (sCr) of 1.70 mg/dL before decompression and 1.35mg/dL after percutaneous nephrostomy tube (PCN) placement. Preoperative median average renal parenchyma thickness (RPT) was 14.5mm. At 6-month follow-up, median sCr and RPT were preserved at 1.25mg/dL (p= 0.084) and 14.3 mm (p=0.41), respectively. At median follow-up of 49.2 months, all patients had a successful repair, defined as no reinsertion of stent/PCN, resolution of hydronephrosis, and no return to the operating room for revision or nephrectomy. Median sCr at last follow-up showed sustained improvement at 1.22 (p=0.0097).

**Conclusions:**

Reconstruction can be successful for obstructed kidneys with less than 20% differential function and may be considered prior to nephrectomy.

## Introduction

Benign ureteral stricture is a relatively rare but potentially devastating disease that can result from urolithiasis, trauma, ischemia, radiation, infection, retroperitoneal fibrosis, iatrogenic causes, or be idiopathic in nature (1). These often present with flank pain but can also remain asymptomatic. The most feared consequence of chronic ureteral obstructions is irreversible renal injury, although urinary tract infections (UTI), hematuria, stone disease, proteinuria, chronic pain, and hypertension may also develop (2, 3). Ideally, strictures are identified early and definitively managed with surgical intervention before permanent damage to kidney function occurs.

Before intervention, ^99m^Tc-mercaptoacetyltriglycine diuretic renography (MAG3 renal scan) is typically obtained if there is a concern for functional loss of the affected kidney (e.g. parenchymal loss on prior diagnostic imaging). In adults, kidneys with a differential renal function (DRF) of<15-20% have traditionally been considered unsalvageable, and nephrectomy has been recommended (3, 4). Endoscopic intervention was shown to be more likely to fail in poorly functioning kidneys by Wolf et al. in 1997 (5), and has been discouraged when DRF is<25% (3, 6). These historical precedents have led to nephrectomy being considered the de facto standard of care in patients with poorly functioning kidneys in this scenario.

With an aging global population and increasing incidence/prevalence of chronic kidney disease, our group now emphasizes maximal renal unit preservation whenever possible (7). This led us to challenge the classic dogma that poorly functioning kidneys are poor candidates for reconstruction. We hypothesize that reconstructive surgery for ureteral strictures in kidneys with less than 20% differential function is safe and successful. Herein, we report our group’s outcomes of ureteral reconstruction in patients with poorly functioning ipsilateral kidneys.

## Materials and methods

### Patient selection

We completed chart reviews of patients who underwent ureteral reconstruction for non-ureteropelvic junction (UPJ) ureteral stricture at our institution between 2013 and 2023. We defined the poorly functioning kidney as a renal unit on the ipsilateral side as the ureteral stricture/obstruction. DRF is established with preoperative MAG3 renal scans and defined for this study as less than 20% ipsilateral function. All patients who underwent ureteral reconstruction with an ipsilateral poorly functioning kidney and had completed a minimum of 6 months of follow-up were consecutively included. Our facility’s institutional review board (IRB) deemed this study to be exempt (IRB Number: Pro00109096).

### Medical/surgical management and follow-up

Prior to surgical intervention for ureteral stricture, our standardized institutional protocol includes: assessment of renal function with a MAG3 renal scan, placement of a percutaneous nephrostomy (PCN) (if not present at the time of referral) with removal of any pre-existing ureteral stent to allow ureteral rest, performance of an antegrade nephrostogram (AN) after 4 to 6 weeks of ureteral rest, review of previously completed renal imaging [ultrasound (US) or computed tomography of the abdomen and pelvis (CT)], and a comprehensive conversation of treatment options (8). Typically, the shared decision-making conversation with patients who have an ipsilateral poorly functioning kidney (defined as<20% DRF on MAG3 renal scan) covers all appropriate surgical intervention for the size and location of stricture, conservative management with chronic PCN/ureteral stent, observation alone, or nephrectomy.

If reconstructive surgery is chosen, patients leave the operating room (OR) with a urethral catheter, ureteral stent, and capped PCN. If the bladder was utilized in the repair, follow-up begins at 10 days with an x-ray cystogram and urethral catheter removal (e.g. ureteral reimplantation bladder elongation psoas hitch (BEPH) and ileal ureter). Catheters are otherwise removed prior to discharge. At 4–6 weeks, AN is repeated and the ureteral stent is removed. After an additional week, AN is again repeated with PCN removal if the ureter is patent and intact. At 6 months post-operation, the patient is seen for symptom review, physical examination, renal function testing, and renal ultrasound. Patients with a history of radiation follow the same care pathway. If the recovery has proceeded without complication, periodic monitoring of renal function and renal imaging can continue with the patient’s primary care provider. Repeat MAG3 renal scans are rarely clinically indicated.

### Data collection and analysis

We recorded patient demographics, resolution of hydronephrosis, need for additional intervention, pre/postoperative renal function, postoperative complications, and preservation of renal parenchyma on imaging. If multiple renal function tests or imaging studies were available, the results nearest to the patient’s initial urology evaluation or follow-up for a given time point were used. Postoperative development or progression of hypertension, flank pain, and recurrent UTI were also recorded. Successful treatment is defined as meeting all the following criteria: no reinsertion of stent/PCN, resolution of hydronephrosis, stability of decompressed renal function, and no return to the OR for revision or nephrectomy by most recent follow-up. The resolution of hydronephrosis was evaluated on both post-operative imaging at 6-month follow-up and the most recent available renal imaging. Most recent follow-up is considered any provider encounter with renal function testing.

Renal function preserved (RFP) is defined as the difference between postoperative estimated glomerular filtration rate (GFR) and obstructed GFR (prior to PCN placement), divided by obstructed GFR, and is reported as a percentage ( $RFP=\left(\frac{GFR_{post\;op}-GFR_{obs}}{GFR_{obs}}\right)x\;100$ ). For example, if a patient with an obstructed kidney has a GFR of 50 ml/min, then undergoes reconstruction rather than nephrectomy and has a documented improvement in GFR to 60 ml/min, RFP would be 20%. To assess the potential effect of compensation by the contralateral (unaffected) kidney, a predicted new baseline glomerular filtration rate (NBGFR) was calculated for each patient using the formula $\text{NBGFR}=1.25\;x\;\left({\text{GFR}}_{\text{Before Intervention}}{\text{ x DRF}}_{\text{Contralateral}}\right)$ . Similar calculations have been described in the nephrectomy literature and are often used in preoperative counseling prior to nephrectomy for malignancy (9).

In conjunction with serum renal function markers (creatinine and GFR), average renal parenchymal thickness (RPT) was measured as a correlate to the function of the renal unit (9–11). Renal volume was not used as it is distorted by preoperative hydronephrosis. RPT measurements were made using the method described by Roger et al. (12) For consistent comparison across the cohort, RPT was measured on preoperative imaging and follow-up imaging at 6-month follow-up. Both US and CT imaging were used for measurements (often only US is obtained postoperatively, while CT imaging more commonly diagnosed the ureteral obstruction), which correlate well enough for this purpose (11).

### Statistical analysis

Statistics were calculated using the Paired T test (two-tailed) and Wilcoxon Signed Rank Test (WSRT). A value of p<0.05 is defined as significant. The two tests agreed on significance unless stated within the text.

## Results

Between 2013 and 2023, 114 patients underwent ureteral reconstruction for non-UPJ ureteral stricture at our institution. Of these, 8 patients had a preoperative MAG3 renal scan showing<20% ipsilateral renal function. Median follow-up was 49.2 months (range: 11.1-93.3 months). All of the patients had follow-up greater than six months; therefore none were excluded from analysis. Patient characteristics are described in **Table 1**. The median DRF of the affected kidney was 16.0% (range: 12.5-19.9%). Half of the cohort failed at least one intervention prior to referral to our center, with 1 patient undergoing both balloon dilation and laser incision and 1 patient undergoing 3 balloon dilations. BEPH was the most common intervention (n=5, 62.5%). All surgeries were performed open.

**Table 1:** Patient characteristics.

Gender, n (%)	
Male	5 (62.5)
Female	3 (37.5)
Age at surgery (years), median (range)	57.1 (44.1-74.7)
Laterality, n (%)	
Right	4 (50)
Left	4 (50)
Stricture Location, n (%)	
Proximal	1 (12.5)
Middle	3 (37.5)
Distal	4 (50)
Stricture etiology, n (%)	
Abdominal surgery	1 (12.5)
Radiation	2 (25)
Urolithiasis/ureteroscopy	5 (62.5)
Differential renal function (%), median (range)	16.0 (12.5-19.9)
Previously failed intervention, n (%)	
Balloon dilation	2 (25)
Laser incision	1 (12.5)
Multiple modalities	1 (12.5)
None	4 (50)
Symptoms, n (%)	
Flank pain	3 (37.5)
Hypertension	1 (12.5)
None	4 (50)
Surgical intervention, n (%)	
BEPH	5 (62.5)
Ureteroureterostomy	1 (12.5)
Ileal ureter + BEPH	1 (12.5)
Ileal ureter	1 (12.5)
Follow-up (months), median (range)	49.2 (11.1-93.3)
Surgery to last renal imaging (months), median (range)	32.6 (11.1-87.5)

BEPH, bladder elongation psoas hitch.

Perioperative and postoperative outcomes are outlined in **Table 2**. Operative times were highly varied (range: 161–513 minutes), with longer procedures associated with operations requiring multiple interventions (ileal ureter with BEPH, 513 minutes; BEPH with abdominal perineal resection, 503 minutes). Reported complications included ileus (n=2, both in patients requiring ileal ureter) and UTI (n=1). No patients required invasive intervention or return to the OR in the postoperative period.

**Table 2:** Patient outcomes.

Operative time (min), median (range)	228 (161-513)
Estimated blood loss (ml), median (range)	100 (100-350)
Hospital length of stay (days), median (range)	3 (1-10)
Complications, n (%)	
Clavien 1-2	3 (37.5)
Clavien ≥3	0 (0)
Postoperative leak (cystogram or nephrostogram), n (%)	0 (0)
Successful reconstruction, n (%)	8 (100)
Preoperative symptoms resolved, n (%)	
Flank pain	3 (100)
Hypertension	1 (100)
Renal parenchymal thickness (mm), median (range)	
Preoperatively	14.5 (10.0-21.0)
Postoperatively (3–6 months)	14.3 (11.0-21.0)
Creatinine (mg/dL), median (range)	
Preoperatively (obstructed)	1.70 (1.0-2.1)
Preoperatively (PCN)	1.35 (1.0-2.1)
Postoperatively (3–6 months)	1.25 (0.9-1.8)
Postoperatively (last follow-up)	1.22 (0.7- 1.72)
Difference obstructed to postop, median (range)	-0.3 (-0.6- -0.1)
Difference PCN to postop, median (range)	-0.1 (-0.5 - -0.1)
Renal function preserved (%), median (range)	24.5 (12.7-37.7)

PCN, percutaneous nephrostomy tube.

Upon follow-up, no patients were found to have a leak on cystogram or nephrostogram, and all urethral catheters/PCNs were removed on schedule. Prior to reconstruction, 3 patients complained of preoperative flank pain, and 1 demonstrated obstruction-associated hypertension. Postoperatively, all three had resolution of their pain and the hypertension normalized. No patients developed new-onset postoperative flank pain, hypertension, recurrent UTI, or proteinuria.

RPT was similar before intervention and at 6-month follow-up at 14.5 mm (range: 10.0-21.0 mm) and 14.3 mm (range:11.0-21.0mm), respectively (p= 0.41). The most recent imaging available was completed at a median 32.1 months (range: 11.0-86.3 months) postoperatively. All patients had a successful reconstruction (as defined above).

**Figure 1:**
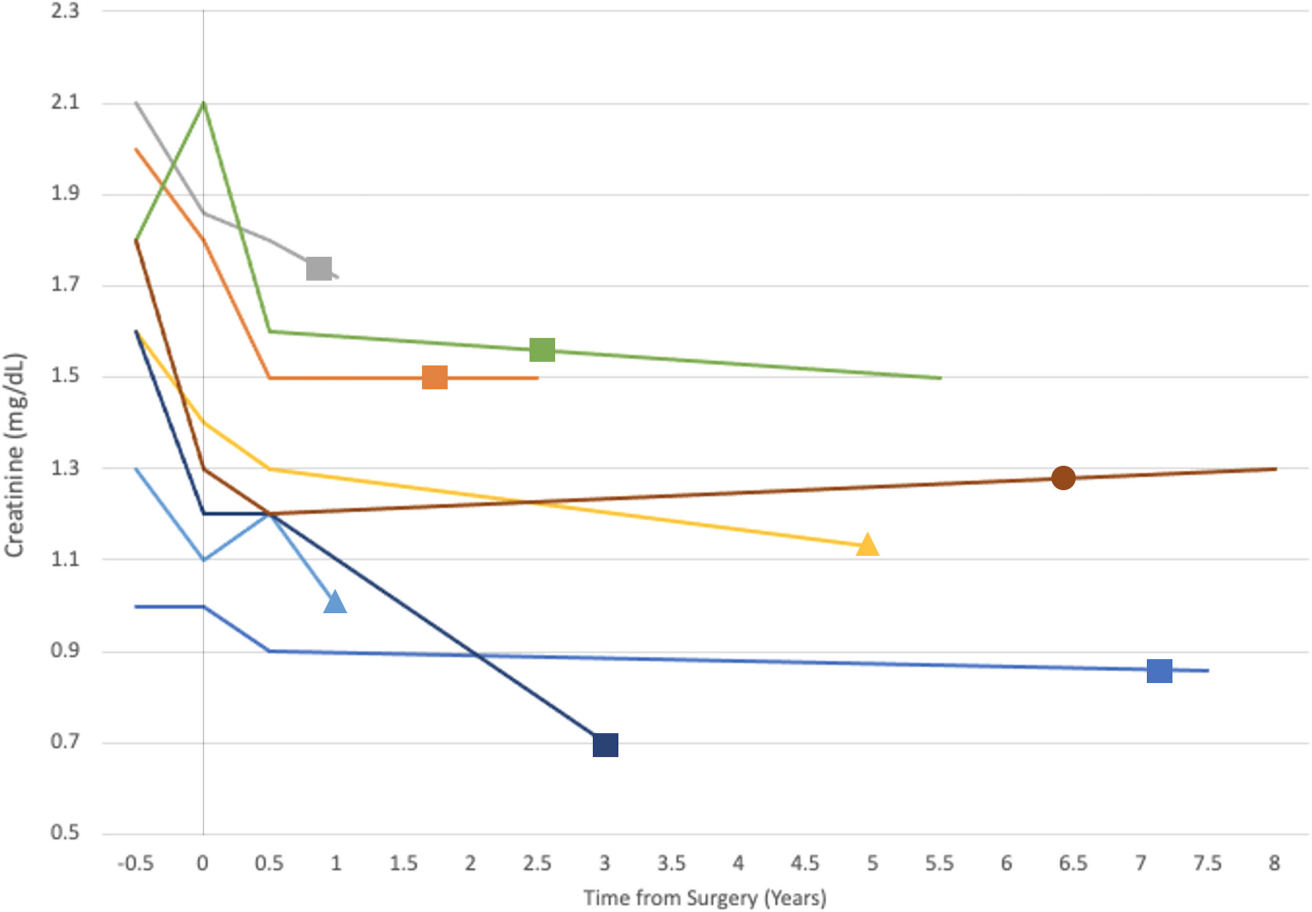
The evolution of creatinine (y-axis) over time (x-axis) in years with 0 on the x-axis marking the time of surgery. Each line represents a single patient and terminates at the time of their last follow-up. Shapes appear on each patient’s line at the time of their most recent renal imaging. Shape type correlates with the surgical intervention undergone by the associate patient: square, bladder elongation psoas hitch (BEPH); triangle, ileal ureter/ileal ureter with BEPH; circle, ureteroureterostomy.

Serum creatinine (sCr) trends are illustrated in **Figure 1**. Prior to reconstruction, renal function improved following PCN placement from a median sCr 1.70 mg/dL (range 1.0-2.1mg/dL) to 1.35 mg/dL (range: 1.0-2.1) (p=0.076). After reconstruction, the median sCr at 6-month follow-up improved to 1.25 mg/dL (range: 0.9-1.8mg/dL) and 1.22 mg/dL (range: 0.7-1.72mg/dL) at the most recent follow-up [p= 0.084, significant on WSRT and 0.0097, respectively (post-PCN sCr compared to follow-up sCr)]. Improvement in sCr between obstructed levels and 6-month follow-up was statistically significant (p= 0.0018). Overall, median RFP was 24.5% (range: 12.7-37.7%), when calculated using GFR at 6-month follow-up. GFR at last follow-up was a median 10.6 ml/min higher (median 24.1% greater) than the predicted NBGFR if nephrectomy was chosen instead of reconstruction (p=0.013).

## Discussion

Attempts to challenge the dogma in adult clinical urology that ureteral obstruction in kidneys with decreased function is best addressed with extirpative surgery have exclusively evaluated UPJ obstruction. This topic has been more thoroughly assessed in pediatric populations, with many studies favoring renal preservation (13–15). A systematic review of pyeloplasty for UPJ obstruction in adults with poorly functioning kidneys showed notable improvement in obstructive symptoms without a clear trend in postoperative renal function across available studies (3 of the 9 included studies showed a statistically significant improvement in renal function, while the others did not show a significant change) (16). Some contemporary examples support higher failure rates of reconstruction in poorly functioning kidneys as originally described by Wolf et al. (5, 17) While no literature is available on the rates of treatment utilization, most poorly functioning kidneys with ureteral stricture are managed with nephrectomy or decompression without attempt at repair. Neither the American Urologic Association nor the European Association of Urology guidelines address this specific scenario. “Poorly functioning kidney” is heterogeneously defined in the literature as less than 10 to 30% differential function. 20% is frequently chosen within this range by UPJ reconstruction investigators and was adopted for this investigation (3, 16). To our knowledge, this is the first report to evaluate the outcomes of ureteral reconstruction for strictures distal to the UPJ in poorly functioning kidneys.

Rising rates of chronic kidney disease (CKD) (incidence increased globally by 88.76% from 1990 to 2016) make nephron-preserving surgical techniques more important than ever before (7). This becomes even more pertinent when considering specific patient populations, including those with cancer, whose treatment options may depend on their GFR. Moreover, renal insufficiency is associated with worse overall survival in patients with solid tumors (18). Urologists have already embraced changes to the surgical standard of care to promote renal preservation. When feasible, partial nephrectomy is a preferred treatment option over radical nephrectomy for this purpose (19). It is imperative to determine if total renal function can be preserved by ureteral reconstruction, even if the ipsilateral kidney contributes<20%.

The incidence of ureteral stricture distal to the UPJ is unknown, but it is generally understood that UPJ obstruction is a more prevalent pathology (3, 4). Pyeloplasty is widely accepted as the gold standard for UPJ obstruction treatment, while management of more distal strictures is less clearly defined and sometimes more technically challenging (20). Given these differences, it is understandable that there is more available literature discussing the management of UPJ obstruction in poorly functioning kidneys (4, 15, 21). It is also likely that few poorly functioning kidneys are being chosen for reconstruction given the widely accepted view that nephrectomy is the preferred treatment (these rates have not been described). Most graduating urology trainees in the United States feel comfortable performing a nephrectomy but may be less experienced with complex ureteral reconstruction (22). In a health system with a shortage of reconstructive urologists, it can be assumed that few kidneys with<20% DRF and ipsilateral ureteral stricture undergo an attempt at reconstruction (23).

Although a distinct disease process from ureteral strictures distal to the UPJ, contemporary literature supports renal preservation in poorly functioning kidneys with UPJ obstruction (14–16, 24). For example, Nishi et al. showed that median improvement in split renal function following pyeloplasty for UPJ obstruction in kidneys with<20% DRF was 24.0% at 6 months and 38.3% at 12 months follow-up (13 of 15 patients in this cohort were adults) (4). Nascimento et al. demonstrated stable renal function following pyeloplasty in kidneys with<15% DRF in a cohort of 15 adult patients (21). While these results would suggest favorable outcomes of ureteral reconstruction for strictures distal to the UPJ in poorly functioning kidneys, they cannot be directly extrapolated.

Despite this cohort’s size, it reveals a statistically and clinically relevant improvement in renal function following ureteral reconstruction. All patients had improved or stable renal function at median follow-up of 49.2 months when compared to the time of their surgery (when decompressed with PCN). The comparison between obstructed renal function and postoperative renal function may be of higher importance. Physiologically, it can be speculated that renal function in a patient with a unilateral chronically, completely obstructed kidney would be similar post-nephrectomy. Median total RFP, which represents the relative change between obstructed and reconstructed total renal function, is 24.5% in this cohort. In other words, if these patients were treated with nephrectomy rather than reconstruction, the predicted median total renal function would be 24.5% lower than the observed postoperative levels.

As observed in patients after nephrectomy, a portion of the improvements in total renal function following ureteral reconstruction could theoretically result from compensation of the contralateral kidney (9, 25). Postoperative MAG3 renograms can evaluate for this confounding factor. The study by Nishi et al. did evaluate pre/postoperative DRF and showed a significant increase in DRF of the affected kidney (as described above) (4). Their findings support the assertion that the reconstructed kidney is contributing to the improvement in overall renal function in a meaningful way. In lieu of postoperative radionuclide imaging in this cohort, analysis of the change in RPT was completed. RPT has been shown to linearly correlate with loss of renal function and CKD (9, 10, 26). It was predicted that stability of RPT in the affected kidney would indicate stability of function, whereas declining RPT would suggest progressive atrophy. No significant change in RPT was noted in this study (median RPT 14.5mm preoperatively and 14.3mm postoperatively). Therefore, it is probable that the affected kidneys continue to have at least a stable DRF. Average RPT varies by region, age, height, weight, and laterality (27). To our knowledge, there are no generalizable reference values for RPT in the literature- significance was only given to individual patients’ RPT trend.

A predicted NBGFR was calculated for each patient to further discern if the reconstructed poorly functioning kidney was contributing to the global renal function independently of contralateral compensation. This NBGFR represents the patients’ expected renal function if their strictures were treated with nephrectomy instead of reconstruction (9). Consistent with the RFP calculations, the cohorts observed GFR was a median 24.1% greater than the predicted NBGFR, which represents a clinically significant difference in renal function. Again, this comparison suggests that a meaningful percentage of total renal function can be preserved with reconstruction of poorly functioning kidneys.

Stability of renal function was of primary interest, but quality of life factors should not be overlooked. Reconstructive surgery offered patients symptom improvement from flank pain (n=3) and hypertension (n=1). All patients were PCN and ureteral stent-free following definitive surgical management, both of which have been shown to have a detrimental impact on lifestyle, but were not specifically evaluated in this study (28).

This study has several limitations inherent to its small sample size and retrospective design. While this is a report on a small patient cohort it is important to remember that for uncommon diseases and rare conditions, case series and reports still contribute significant value to the literature (29). Each individual intervention in this cohort was not uniform amongst all patients making direct comparison somewhat difficult. Treatment choice for ureteral stricture is nuanced and ureteral stricture reconstruction in poorly functioning kidneys is rare, which makes the organization of a sizable or homogenous cohort difficult. Selection bias is inherent to retrospective studies; patients with a larger number of comorbidities or those requiring more difficult reconstructions may have undergone conservative management or nephrectomy outside of our healthcare system without referral. In this case, our cohort would comprise better surgical candidates who would be expected to have preferable outcomes. Given our experience, we do not believe that a randomized controlled trial would be possible. However, a multi-institutional case-control study would offer higher quality evidence to support the efficacy of reconstruction of kidneys with<20% DRF and should represent the next step of future research. The findings described here may not single-handedly change the current treatment paradigm, but they do demonstrate that ureteral reconstruction in poorly functioning kidneys can be completed safely and successfully.

In conclusion, upper urinary tract reconstruction was safe and successful for obstructed kidneys with less than 20% split function within this small patient series. While more robust investigation is indicated, we recommend abandoning the dogma of nephrectomy alone for these patients to maximize the preservation of renal function.

## Data Availability

The raw data supporting the conclusions of this article will be made available by the authors, without undue reservation.
